# Miniaturization of an Osmotic Pressure-Based Glucose Sensor for Continuous Intraperitoneal and Subcutaneous Glucose Monitoring by Means of Nanotechnology

**DOI:** 10.3390/s23094541

**Published:** 2023-05-07

**Authors:** Andreas Pfützner, Barbora Tencer, Boris Stamm, Mandar Mehta, Preeti Sharma, Rustam Gilyazev, Hendrick Jensch, Nicole Thomé, Michael Huth

**Affiliations:** 1 Lifecare AS, 5058 Bergen, Norway; barbora.tencer@lifecare.no; 2 Lifecare Nanobiosensors GmbH, 55128 Mainz, Germany; boris.stamm@lifecare.no (B.S.); mandar.mehta@lifecare.no (M.M.); preeti.sharma@lifecare.no (P.S.); rustam.gilyazev@lifecare.no (R.G.); 3 Lifecare Laboratories GmbH, 55128 Mainz, Germany; hendrick.jensch@lifecare.no (H.J.); nicole.thome@lifecare.no (N.T.); 4 Pfützner Science & Health Institute, 55128 Mainz, Germany; 5 Institute for Internal Medicine and Laboratory Medicine, University for Digital Technologies in Medicine & Dentistry, 9516 Wiltz, Luxembourg; 6 Institute of Physics, Goethe-Universität, 60323 Frankfurt am Main, Germany; michael.huth@physik.uni-frankfurt.de

**Keywords:** continuous glucose monitoring, osmotic pressure, NTR sensor, FEBID, implantable glucose sensor

## Abstract

The Sencell sensor uses glucose-induced changes in an osmotic pressure chamber for continuous glucose measurement. A final device shall have the size of a grain of rice. The size limiting factor is the piezo-resistive pressure transducers inside the core sensor technology (resulting chamber volume: 70 µL. To achieve the necessary miniaturization, these pressure transducers were replaced by small (4000 × 400 × 150 nm³) nano-granular tunneling resistive (NTR) pressure sensors (chamber volume: 750 nL). For benchmark testing, we filled the miniaturized chamber with bovine serum albumin (BSA, 1 mM) and exposed it repeatedly to distilled water followed by 1 mM BSA solution. Thereafter, we manufactured sensors with glucose testing chemistry (ConcanavalinA/dextran) and investigated sensor performance with dynamic glucose changes between 0 and 300 mg/dL. Evaluation of the miniaturized sensors resulted in reliable pressure changes, both in the BSA benchmark experiment (30–35 mBar) and in the dynamic in vitro continuous glucose test (40–50 mBar). These pressure results were comparable to similar experiments with the previous larger in vitro sensors (30–50 mBar). In conclusion, the NTR pressure sensor technology was successfully employed to reduce the size of the core osmotic pressure chamber by more than 95% without loss in the osmotic pressure signal.

## 1. Introduction

Measuring glucose levels is part of the multiple daily routine procedures for patients with diabetes mellitus on insulin therapy. On the basis of the measurement results, millions of therapy decisions (e.g., regarding the insulin dose to be injected) are made every day worldwide, which can have an influence on the patient’s short-term and also long-term well-being [1]. In addition to the spot glucose measurements, various systems for continuous glucose measurement (CGM) have become available in the last two decades. CGM systems are devices that measure glucose levels in interstitial fluid in the subcutaneous tissue based on glucose-oxidase measurement technology by means of needle-based sensors; usually, the measurement result is provided every five minutes and transmitted to a receiving handheld or smartphone. The first CGM device was introduced by MiniMed at the beginning of the millennium. It proved to be functional for three days and needed to be calibrated several times during the day to have acceptable accuracy [2]. Since then, technology has improved massively, and better measurement accuracy and prolonged duration of use have made CGM with needle sensors a frequently used diagnostic tool for diabetes management of all patients with type 1 and also of many of those with type 2 diabetes mellitus [1]. 

CGM systems provide users with much more data about their glucose course, which also enables the usage of different parameters to characterize this (e.g., Time-in-Range [3]). In addition, specific threshold values can be defined, and an alarm is triggered if glucose levels exceed these cut-offs. The use of CGM systems has hence resulted in a reduced frequency of hypoglycemic and hyperglycemic events, an overall improvement of glycemic control, and an improved quality of life [4,5,6,7,8,9,10,11,12,13]. However, the current glucose sensors need to be replaced every 10 to 14 days, and they have interference and accuracy issues [14,15,16]. In consequence, there is still a medical need for the development of small glucose sensors with improved measurement properties. Ideally, such sensors would be implanted and can be used for long time periods. They should be economically affordable and should not have a significant environmental impact, as usage of current CGM systems generates a lot of (plastic) waste. 

One approach to reach this goal is the development of Sencell (Lifecare AS, Bergen, Norway), a small and implantable glucose sensor employing wireless energy and data transfer. This sensor utilizes osmotic pressure changes in a closed chamber induced by increasing and decreasing glucose concentrations in the interstitial fluid outside of the device. The underlying osmotic chamber technology was presented a decade ago by Johannesen et al. [17]; however, their prototype device was too large (5 × 5 × 2 cm³) and had a too slow response time (45–60 min) to glucose changes to be clinically useful.

The aim of the current development is a glucose sensor with the size of a rice grain, a response time of maximally a few minutes, and a duration of use of at least 6–12 months. The final sensor product is planned to be implanted in the subcutaneous tissue at the forearm underneath a watch-like readout unit. The watch will operate the sensor with an inductive power supply and a wireless readout. In case of the necessity to remove the device from the tissue (e.g., at the end of its operations), appropriate (proprietary) means for an easy location and removal with a minor surgical procedure will be considered. The development steps undertaken to reach the actual size of the core sensing unit (1 × 0.5 × 0.25 mm) are described.

## 2. Materials and Methods

### 2.1. Measurement Technology 

The underlying osmotic pressure technology for the continuous glucose measurement sensor was published more than a decade ago [17]. In brief, an active fluid with a large glucose-binding molecule (GBM) and a large glucose-like ligand (GL) are present in a closed chamber. This is exposed to a fluid with different glucose concentrations and generates measurable osmotic pressure signals. In a glucose-free solution, the GBM and the GL form a complex because of the electrostatic binding of the GL to the GBM at a glucose-specific binding site. When glucose penetrates through a semipermeable (size exclusion) membrane into the chamber, the GL will go off from the GBM binding sites, as glucose has a slightly higher binding affinity to the GBM receptor. Subsequently, every GL molecule freed from the GBM-GL complex enhances the osmotic pressure inside the chamber. This reaction is fully reversible, and decreasing glucose concentrations on the outside makes glucose molecules leave the GBM binding site. This allows GBM and GL to reassemble, and the osmotic pressure declines. There is a linear relationship between the glucose concentration in the external fluid and the measurable osmotic pressure in the chamber [17]. The described reversible affinity reaction in the chamber does not consume any molecules when generating the signal, providing the potential for long-term usage of the sensor in the body. 

**Figure 1:**
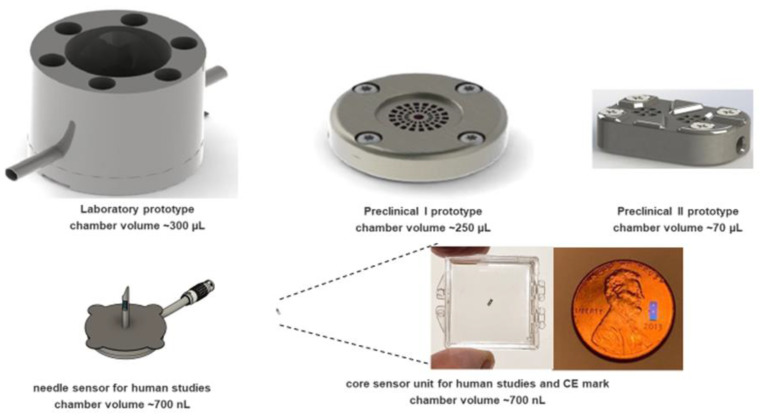
Preclinical prototypes using piezo-resistive pressure transducers (upper line) and the miniaturized versions employing the core sensing unit with NTR sensors on the nanoscale (lower line).

In the last years, wired in vitro prototypes based on this sensing technology have been developed. The most current version of these used the smallest commercially available piezo-resistive (PR) pressure transducers to determine the glucose-induced osmotic pressure changes, resulting in a chamber volume of 70 µL (Figure 1). 

**Figure 2:**
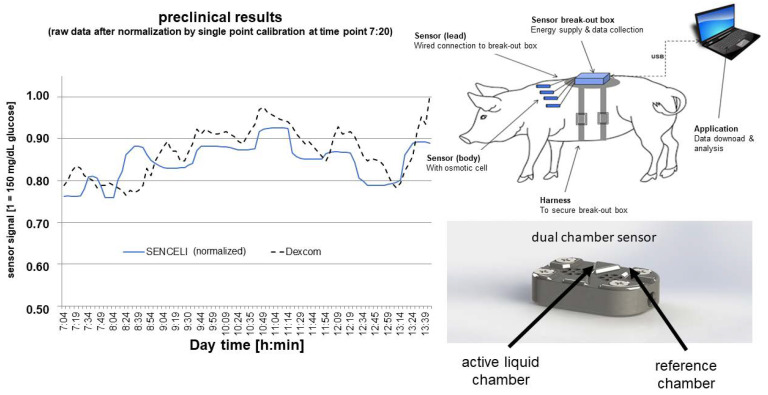
Sketch of the animal study setup and an example for the measurement results after one-point calibration in comparison to a commercially available CGM system.

These devices were used for in vitro experiments and for preclinical proof-of-concept studies in pigs. A sketch of the experimental animal study setup and an example of the performance results obtained with these prototypes are provided in Figure 2. 

However, the prototypes needed to be further miniaturized to be clinically useful. At this stage, it was not possible to further reduce the size of the sensor chamber because further miniaturization of the (conventional) PR pressure transducers was not possible without losing the necessary sensitivity for pressure sensing; a novel measurement principle was needed. 

### 2.2. Nano-Granular Tunneling Resistive (NTR) Sensors

In 2016, Dukic et al. published a report about the manufacturing process and the physico-chemical properties of pressure-sensing elements with a size in the nanometer range [18]. The sensors were built by means of focused electron beam-induced deposition (FEBID) using a direct-write technology (Nano3Dsense^®^, Nanoscale Systems, Darmstadt, Germany). In brief, an electron microscope was modified with a gas injection system. An electron beam was directed to the spot of the planned sensor location. From the side, a metal-organic precursor gas flow was directed to the same spot. In the focus of the electron beam, previously adsorbed precursor molecules were dissociated, resulting in a permanent deposit. The deposit microstructure is that of a nano-granular metal if the precursor gas species is properly chosen [19]. The final size and the structure of the “printed” product are defined by the software-controlled process and can be adapted as desired. 

**Figure 3:**
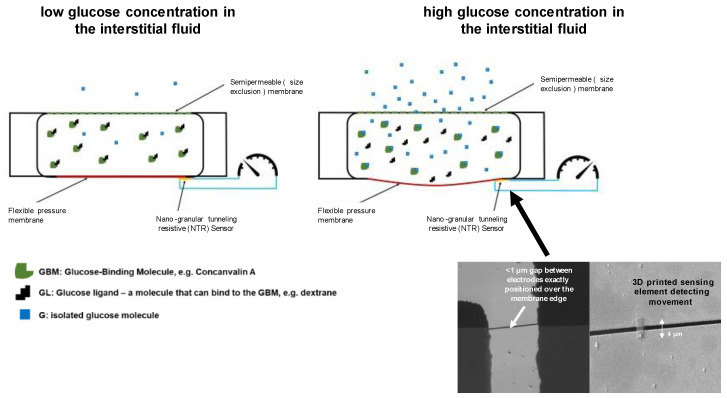
Sketch of the osmotic pressure-based glucose sensor indicating the measurement conditions at low and high glucose concentrations in the interstitial fluid.

In the most current glucose sensor, the pressure sensor is located at the base of a membrane that forms the bottom of the chamber opposite to the upper part, which is covered by the semipermeable membrane (Figure 3). It closes a small gap between two gold electrodes. A pressure increase results in movement and straining of the pressure membrane causing a resistivity change in the sensor element, which is part of a Wheatstone bridge. This sensing technology allowed for miniaturization of the osmotic pressure chamber to a volume of 750 nL and to reduce the operating voltage to 100 mV.

For the in vitro and preclinical in vivo experiments, the signal from the sensor system was directly read out by a voltmeter (Keithley DMM6500, Tektronix, Beaverton, OR, USA). The sensor system did not use a pre-amplifier and was operated with a stable 100 mV low-noise voltage regulator system based on an LT3042 chip for analog devices. The recorded data was saved via a self-written Python code, which also provided a separate window for real-time data visualization. After the experiment, the data was stored as a standard CSV file and was further processed with standard analysis software. 

## 3. Results

### Osmotic Pressure Benchmark Testing

**Figure 4:**
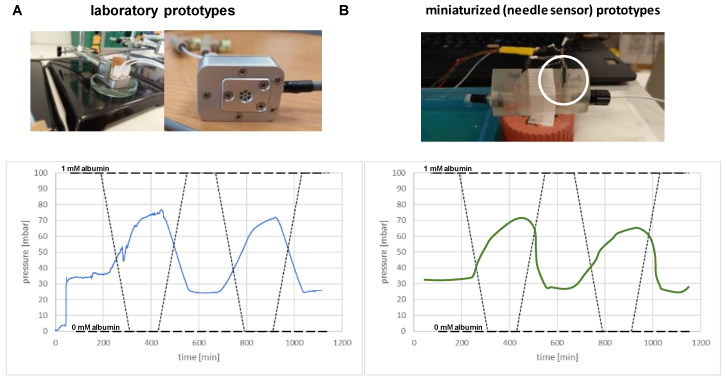
Osmotic pressure changes induced by exposure of the sensors filled with 1 mM bovine albumin solution to distilled water and 1 mM BSA solution. (**A**) Results with the laboratory prototypes using piezo-resistive pressure transducers, and (**B**) results with the miniaturized core sensing unit employing NTR sensors.

The functionality of such in vitro sensor prototypes with piezo-resistive pressure sensing was first assessed in a benchmark testing experiment, such that the chamber was filled with 1 mM albumin in a physiological NaCl solution. Exposing these sensors to distilled water had previously led to a pressure increase by ~30 mBar, and consecutive exposure to 1 mM albumin solution resulted in a pressure decrease back to baseline (Figure 4A). It is well established that osmotic pressure is independent of the chamber volume, and using the same protocol, the miniaturized albumin-containing sensors provided similar results when exposed to the same series of solutions (Figure 4B). All experiments were repeated at least in triplicate confirming this outcome.

**Figure 5:**
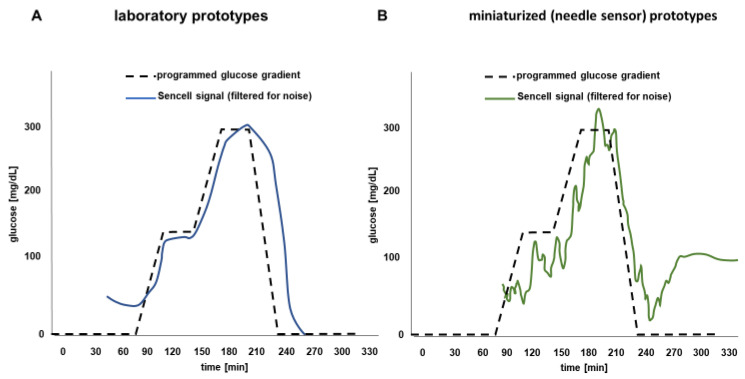
Osmotic pressure changes induced by exposure of the sensors filled with an active 1.5 mM Concanavalin A/dextran solution when exposed to 2 mM and 30 mM glucose concentrations in physio in solution to distilled water and 1 mM BSA solution. (**A**) Results with the preclinical prototypes using piezo-resistive pressure transducers, and (**B**) results with the miniaturized core sensing unit employing NTR sensors for pressure sensing.

Based on these results, the miniaturized sensors were now manufactured with inclusion of the active glucose-sensing Concanavalin A/dextran chemistry [17]. When exposing these sensors to dynamic glucose concentration in the fluid around the sensors, changes by using an in vitro dynamic CGM test rig [16], similar osmotic pressure changes were seen as with the previous larger prototypes (Figure 5). Pressure changes were reproducible, and a linear relationship between the sensor signal and varying glucose concentrations in the supernatant was observed. 

The results of the miniaturized sensors (Figure 5B) show that the signal-to-noise ratio becomes a bit weaker proportional to the size of the pressure membrane resulting in a more pronounced variability of the current readout. The increased signals at an (unphysiological) glucose concentration of 0 mg/dL in the beginning (A) or at the end (B) of the experiments, respectively, are artifacts induced by the employed standard smoothening filter, which acts over frequencies (Butterworth filter). An optimized algorithm to address such artifacts will be developed once human clinical results become available. 

**Figure 6:**
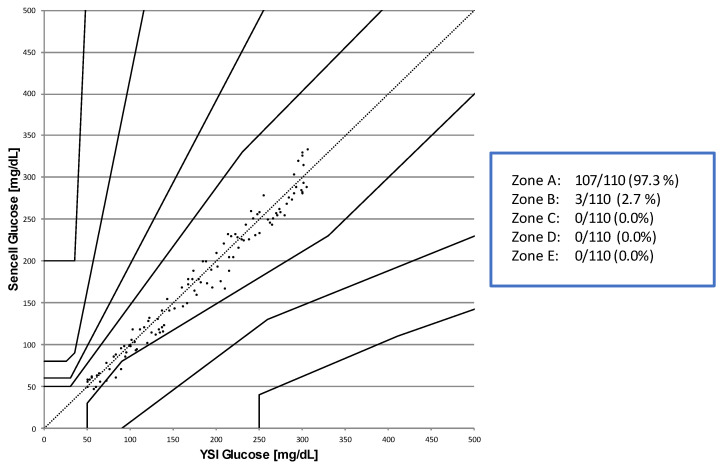
Consensus error grid analysis of the in vitro data derived from the miniaturized osmotic pressure-based sensor in comparison to a reference method (YSI STAT2300 Analyzer, *n* = 150).

The delay between the employed and measured glucose concentrations in the in vitro experiments is induced by the time required to deliver the programmed glucose levels through the tubes from the pump to the sensors. After correction for this time shift, a Parkes-Error-Grid analysis [20,21] (vs. YSI STAT2300 Plus Glucose Analyzer as a reference method, YSI Inc., Yellow Springs, OH, USA) for the clinically relevant glucose ranges (50 to 300 mg/dL) confirmed that 100% of the data points were in the non-critical zones A + B in this in vitro consensus error grid, which is e.g., a regulatory clinical performance requirement for glucose monitoring devices for prescribed point-of-care use [22]. The results of this analysis are provided in Figure 6.

## 4. Discussion

The principle of osmosis and the use of osmotic pressure to stabilize biological structures, e.g., biological cells, is a common phenomenon in nature. As can be seen from its formula—π = I × M × r × T (where “π” is osmotic pressure, “I” is the van’t Hoff factor, “M” is the molar concentration of the particles in solution, “R” is the ideal gas constant, and “T” is the temperature in Kelvin)—osmotic pressure does not depend on volume. In theory, the core sensing technology could hence be miniaturized to a size in the 100-nanometer range without loss of the general efficacy and sensitivity. However, there are several reasons suggesting that it may be better to not push the miniaturization potential to its extremes, but still be smaller than the size of existing CGM systems in the market. Firstly, an injectable glucose sensor must still be visible and easy to handle to be inserted in the physician’s office. Secondly, space is required to attach an ASIC (integrated electronic circuit with tailored functionality) for energy induction and data transfer to a readout unit. Thirdly, the manufacturing process must allow the handling of the sensors during a fully automated mass production process, which includes the filling of the osmotic pressure chamber with the glucose sensing fluid and sealing of the chamber. Furthermore, it may still occasionally be required to remove a non-functional sensor from the implantation site at a later stage. In such a case, the device geometry needs to be adequate so this can be accomplished easily. Finally, the measurement process for measuring osmotic pressure changes must be reliable under all circumstances, e.g., changes in environmental pressure or ambient temperature. 

While osmotic pressure—as stated above—is independent of volume and is the same in a 100 L barrel and in a human cell, as long as the temperature and fluid compositions (molecule type and concentration) inside and outside of the structure carrying the volume are identical, a size-limiting factor is the method used for translation of the osmotic pressure change into an electrical signal. In the current glucose sensor version, pressure sensing has become a key factor in the miniaturization of the sensor. Piezo-resistive pressure transducers cannot be miniaturized in a similar way as the chamber size (by 99% from 50–70 µL to 500–700 nL). Such transducers lose sensitivity when going below a certain size [23,24], which limits their usability in glucose sensor development. This situation forced a search for alternative pressure-sensing options. A comprehensive literature research helped to identify NTR sensors as a very compelling alternative to achieve the desired overall small glucose sensor size of a grain of rice. This nanotechnology approach has several advantages:

The NTR structures can easily be manufactured on a wafer-based large scale by means of fully automated focused electron beam-induced deposition (FEBID) using a Nano3DSense 3D printing process, which allows using multi-beam electron microscopes. In addition, the sensors can be printed on any material. The required operation voltage in the range of 100 mV or below can easily be delivered by several wireless energy induction technologies. Finally, the technology and the associated electronic requirements allow using existing and commercially available solutions for wireless data transfer. In addition, the results can be displayed in a user-friendly manner, e.g., by an App on a smartphone. 

The development of implantable sensors or devices based on non-invasive methods for the determination of blood or tissue glucose has become a major goal in diabetes technology during the last decades. The physical methods used to assess specific glucose signals in blood, tissue, saliva, retinal fluid, and other organs include but are not limited to (near/mid) infrared spectroscopy, photoacoustic spectroscopy, terahertz spectroscopy, Q_u_—Based Resonant Microwave sensing, Raman spectroscopy, radio impedance spectroscopy, optical rotation, and even combinations thereof [25,26,27]. Still, non-invasive devices need a periodically invasive calibration. Similar to the minimally invasive needle sensors, the information yielded by the applied sensors can be delayed (15–20 min) as blood glucose needs to be shifted to the interstitial tissue first, which limits the accuracy of devices in emergency situations [25]. Based on the underlying measurement technology, non-invasive devices may also suffer from additional sources of interferences, including differences in skin properties, alterations in microcirculation and individual blood supply, current medication, and comorbidities. For these and other reasons, most of the non-invasive technologies have so far not met the required standard of accuracy. However, with the rapid development of wearable technology and transdermal biosensors, non-invasive blood glucose monitoring is likely to become more efficient, affordable, robust, and more competitive on the market [28].

Currently, there is one implantable glucose sensor available on the market, which is approved to be used for up to 90 days (in the US) or up to 180 days (in the EU), respectively (Eversense, Senseonics, Germantown, MD, USA). The sensor measures the glucose concentration in interstitial fluid every five minutes and displays the measurements on a smartphone [29]. The sensor works with fluorometric glucose assessment and has a size of 3.3 mm (diameter) × 15 mm (length). It is implanted in the subcutaneous tissue of the upper arm using local anesthesia by a trained healthcare professional. However, the sensor signal has a general drift and requires a conventional blood glucose measurement for calibration approximately every 12 h in order to accurately measure glucose (MARD 8.5% to 11.5%). The CGM system is designed to replace blood glucose measurements for diabetes treatment decisions, but it needs to become more robust in the measurement performance to ultimately reach this goal [30,31,32,33]. The sensors need to be removed after maximally 6 months of use, which requires a skin incision and dissection to identify the sensor in the subcutaneous tissue and its surrounding fibrous capsule. This can be a difficult procedure, in particular when the sensor has migrated through the tissue during use and may require assistance from a surgeon [29]. The benefits of this implantable system may exceed the short-term discomfort following implantation and the small risk of infection, hematoma, skin irritation, and premature sensor failure [29]; however, further efforts are needed to improve the performance of this device. There is clearly a medical need for alternative glucose measurement technologies, which is an encouragement to continue with the osmotic pressure-based sensor development described before.

In conclusion, miniaturization of the core sensing element of an osmotic pressure-based continuous glucose sensor to meet the requirements for the planned size of the entire injectable device was achieved by replacing the former piezo-resistive pressure transducers with NTR sensors with a size in the 100 nm range. During the clinical development process, wired versions of the core sensing unit have been built into small needles and are currently subject to the first clinical experiments in human volunteers. 

## Data Availability

The original data of the experiments described in this article is on file at Lifecare A/S.
